# Case Report: Multimodal imaging of a macular vortex vein in a highly myopic eye

**DOI:** 10.3389/fmed.2026.1841332

**Published:** 2026-06-19

**Authors:** Zhilin Zhang, Shuang Wang, Xingyu He, Keke Huang, Ting Luo

**Affiliations:** Department of Ophthalmology, The Third People’s Hospital of Chengdu, Southwest Jiaotong University, Chengdu, Sichuan, China

**Keywords:** case report, high myopia, macular vortex vein, multimodal imaging, swept-source optical coherence tomography angiography

## Abstract

**Introduction:**

Macular vortex veins are a rare anatomical variant that can occur in highly myopic eyes. Their pathogenesis remains controversial, with some studies suggesting a congenital origin and others proposing acquired changes secondary to myopic progression. The presence of posterior staphyloma has been closely associated with these venous anomalies. Here, we report a case of macular vortex vein in a highly myopic eye, evaluated using multimodal imaging.

**Case presentation:**

A 51-year-old myopic female presented with a 2-week history of floaters in the right eye. She denied any prior ocular history other than refractive error, as well as any systemic chronic diseases or ocular trauma. Best-corrected visual acuity was 20/20 in the right eye and 20/25 in the left eye. Myopia was −6.5 D in the right eye and −6.75 D in the left eye. Ultra-widefield color fundus photography revealed dilation of the large choroidal vessels in the macular region of the left eye, with a prominent vortex vein ampulla. Infrared reflectance imaging clearly demonstrated the vortex vein branches converging toward the macula. Indocyanine green angiography (ICGA) showed centripetal convergence, well-defined contours, and unobstructed drainage of the macular vortex veins. Swept-source optical coherence tomography angiography (SS-OCTA) further revealed the medium-to-large choroidal vessel architecture. En face OCTA of the large-vessel choroidal slab clearly delineated the luminal structure of the macular vortex veins. Cross-sectional B-scans with flow overlay demonstrated the morphology of the vortex vein lumen.

**Conclusion:**

Macular vortex veins can occur in highly myopic eyes. Multimodal imaging, especially SS-OCTA, helps visualize this vascular variant clearly. Recognizing this benign finding is important to avoid misdiagnosis and unnecessary treatment.

## Introduction

The vortex veins constitute the main venous drainage system of the choroid. Their ampullae are visible ophthalmoscopically and typically serve as a landmark for the equator (1, 2). In rare cases, vortex veins exhibit anatomical variations and are located in the posterior pole, collectively referred to as posterior vortex veins (PVVs). Based on their exit sites, Moriyama et al. classified PVVs into five types: parapapillary type, macular type, peripapillary staphyloma edge type, macular atrophy or large peripapillary atrophy edge type, and other regions type, among which the macular type accounted for approximately 17% (3).

The pathogenesis of PVVs remains controversial. One hypothesis suggests that these structures may represent an acquired change in highly myopic eyes, with a reported detection rate of 11–26.4% in this population (3–5). Other studies have observed PVVs in healthy eyes, implying a possible congenital origin. He et al. used wide-field optical coherence tomography angiography (wide-field OCTA) to examine the presence of PVVs. The results showed that such vascular structures were detectable in 16.1% of healthy eyes, 6.1% of which were located in the macular region. Furthermore, the detection rate of PVVs increased with the severity of myopia: 10.3% in emmetropic eyes, 16.6% in low-to-moderate myopic eyes, and 26.4% in highly myopic eyes (6). These findings suggest that PVVs may be congenitally present but become more apparent under the influence of refractive status. Evidence for such a congenital basis can also be found in case reports of several congenital developmental disorders. For example, the presence of PVVs has been observed in patients with trisomy 13 syndrome (7), Donnai-Barrow syndrome (8), and oculocutaneous albinism (9).

Swept-source optical coherence tomography angiography (SS-OCTA) offers depth-resolved and non-invasive imaging, enabling simultaneous evaluation of choroidal vascular morphology and blood flow characteristics, thus providing a powerful new tool for investigating PVVs (6).

This study reports a case of macular vortex vein in a highly myopic eye using multimodal imaging, including indocyanine green angiography (ICGA) and SS-OCTA. The patient has normal binocular visual acuity, with an incidental macular vortex vein detected in the left eye and no abnormalities in the fellow eye. We present a detailed multimodal imaging description of this vascular anomaly and discuss its unilateral occurrence and interocular asymmetry, providing new evidence and insights into the pathogenesis of PVVs.

### Case presentation

A 51-year-old myopic female presented with a 2-week history of floaters in the right eye. She denied any prior ocular history other than refractive error, as well as any systemic chronic diseases or ocular trauma. At the initial visit, her best-corrected visual acuity was 20/20 in the right eye and 20/25 in the left eye. Myopia was −6.5 D in the right eye and −6.75 D in the left eye. The patient’s myopia started at age 10 and progressed quickly through adolescence. It stabilized after she became an adult, with no significant recent changes. Her family history for high myopia is negative. Axial length measurements (IOL Master 700; Carl Zeiss Meditec AG, Jena, Germany) were 27.98 mm in the right eye and 27.94 mm in the left. Anterior segment examination was unremarkable.

**Figure 1:**
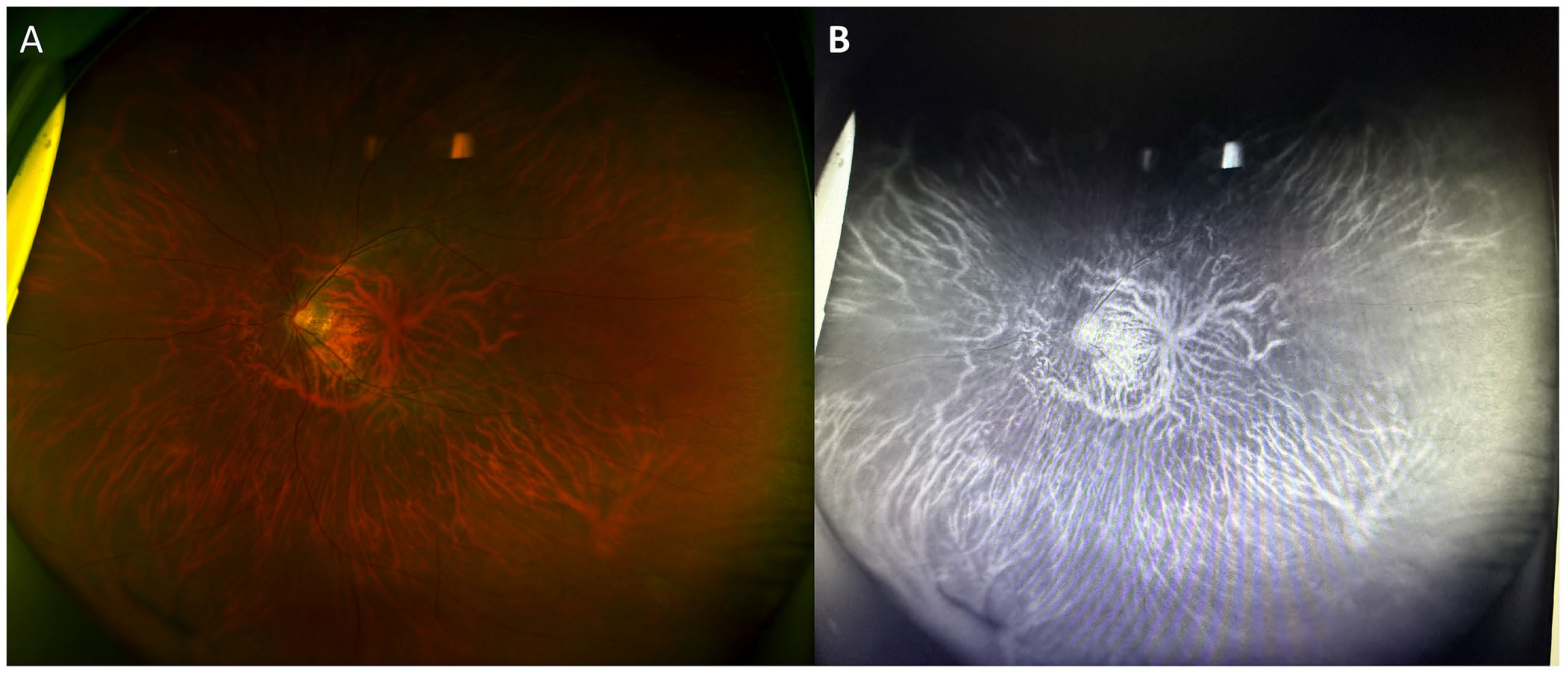
Ultra-widefield fundus photograph and infrared image. **(A)** Fundus photograph showing a macular vortex vein. **(B)** Corresponding infrared reflectance image clearly showing the vortex vein converging in the macular area.

Ultra-wide-field color fundus photography (Optos® 200Tx; Optos PLC, Dunfermline, UK) showed a macular vortex vein in the left eye (Figure 1A). Infrared reflectance imaging clearly demonstrated the vortex vein converging in the macular area (Figure 1B).

**Figure 2:**
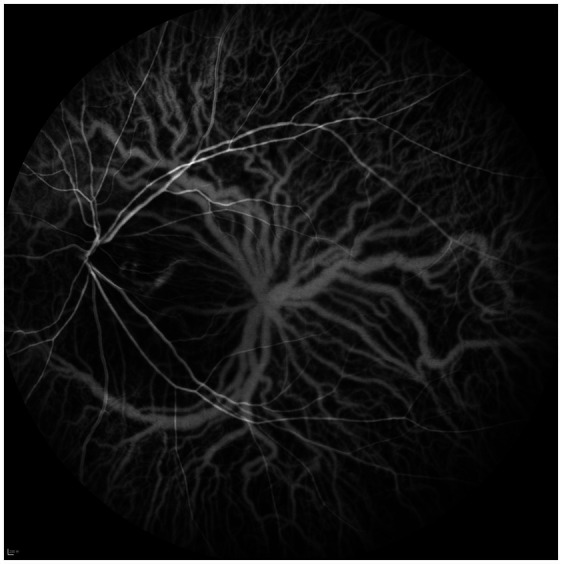
ICGA image of macular vortex veins. ICGA image showing the macular vortex veins with centripetal convergence, well-defined contours, and unobstructed drainage.

ICGA (HRA Spectralis; Heidelberg Engineering, Heidelberg, Germany) showed centripetal convergence, well-defined contours, and unobstructed drainage of the macular vortex veins (Figure 2).

**Figure 3:**
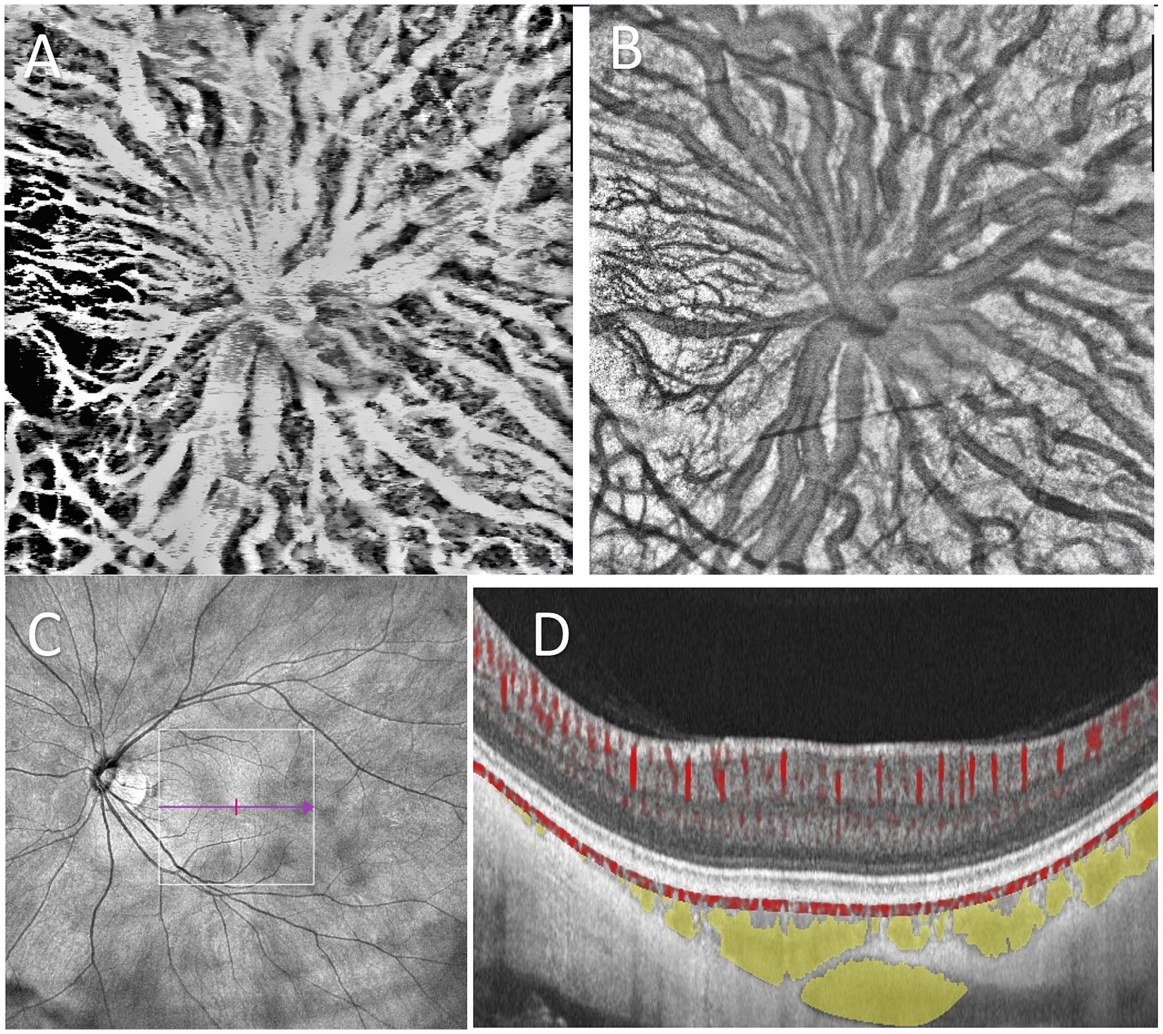
Multimodal imaging of macular vortex veins. **(A)** 6.00 × 6.00 mm wide-field-OCTA in the large-vessel choroidal layer showing medium-to-large choroidal vessels in the macula. **(B)** en face wide-field-OCTA image showing the medium-to-large-vessel choroidal layer in the same scan as A. **(C)** Cross-sectional OCT B-scan fundus image showing the location of the 12.00 mm scan line. **(D)** Corresponding OCT B-scan with angiographic flow overlay. The luminal contour of the choroidal vortex vein is delineated in yellow.

SS-OCTA (TowardPi BMizar; TowardPi Medical Technology, Beijing, China) further revealed the medium-to-large choroidal vessel architecture. En face OCTA of the large-vessel choroidal slab (6.00 mm × 6.00 mm) clearly delineated the luminal structure of the macular vortex veins (Figures 3A,B). Cross-sectional B-scans with flow overlay demonstrated the morphology of the vortex vein lumen, with its contour outlined in yellow (Figures 3C,D).

**Figure 4:**
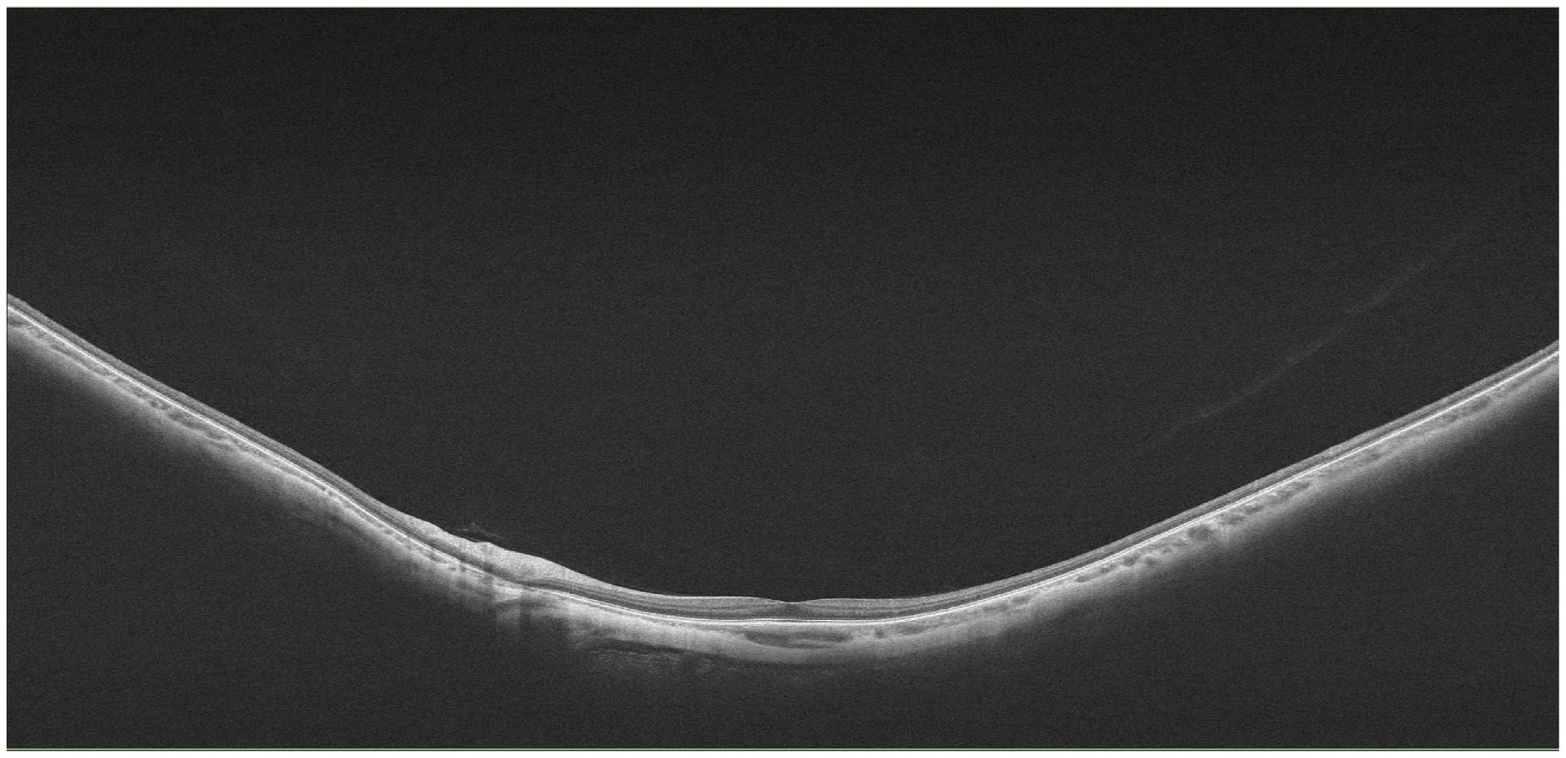
Ultra-widefield SS-OCT B-scan. The scan (26 mm) shows a smooth posterior pole contour without obvious staphyloma. Mild choroidal thinning is noted on the nasal side of the optic disc.

Ultra-widefield SS-OCT B-scan (TowardPi BMizar; Figure 4) was performed with a scan width of 26 mm to show a smooth posterior pole contour without obvious staphyloma. Mild choroidal thinning was noted on the nasal side of the optic disc. Given the incidental nature of the finding, the patient was reassured of the benign variant, advised for annual follow-up, and no intervention was required.

## Discussion

The macular vortex vein was comprehensively evaluated using fundus photography, ICGA, and SS-OCTA. The B-scan of SS-OCTA clearly demonstrated a dilated hyporeflective lumen within the Haller’s layer of the choroid, while en-face images vividly illustrated its tortuous, spider-like configuration and drainage pattern. Compared with the surrounding choroidal stroma, the vessel showed a prominent relative flow void sign, consistent with the OCTA features first described by Kaplan et al. (10). This hypoperfusion state corresponds to its function as a passive venous outflow channel.

The association between posterior staphyloma and PVVs has been highlighted in several studies. Moriyama et al. (3) found the prevalence of posterior staphyloma was significantly higher in eyes in which PVVs were detected than in eyes without PVVs, and categorized a subset of PVVs as “staphyloma edge type” (6%). In a separate study, the same group observed marked structural alterations in choroidal vessels in highly myopic eyes, which were more prevalent in eyes with posterior staphyloma (5). The presence of PVVs in the absence of staphyloma suggests that additional mechanisms may be involved. Ohno-Matsui et al. (4) proposed early on that macular vortex veins might exist in healthy eyes but remain functionally inactive and undetectable; altered choroidal blood flow in highly myopic eyes could lead to dilation and thus visualization of these veins. The absence of a typical, definitive staphyloma in our case supports this view and reinforces the congenital anatomical variation hypothesis. Nevertheless, as described by Shinohara et al. (11), the mild choroidal thinning and subtle inward scleral protrusion noted on the nasal side of the optic disc may represent early signs of posterior staphyloma formation.

Moriyama et al. (3) described five PVV types. The type that drains along the staphyloma edge is rare—only 6% of cases. When a PVV and posterior staphyloma coexist (especially this type), the tortuous vessels exit at the steep edge. That can make them easy to miss or misdiagnose. The same study also found that choroidal venous flow can completely stop at that steep edge, leading to sluggish flow and collateral channels—which adds to the complexity. Another finding: 17.5–21% of PVV eyes had CNV-related macular atrophy, with the vein exiting right next to the atrophy edge. So when a PVV and staphyloma coexist, the risk of misdiagnosis (especially with myopic CNV) is real. In such cases, multimodal imaging is key. No leakage on ICGA plus a flow void on OCTA can reliably tell a benign PVV from active CNV (3, 10). In our case, we did not have a typical staphyloma, but we used the same imaging approach and confirmed the macular vortex vein as a benign incidental finding. That saved the patient from unnecessary treatment.

ICGA has been widely recognized as the gold standard for imaging choroidal vasculature. However, it has notable disadvantages, including invasiveness, long examination time, and potential risks of adverse events (12).

Optical coherence tomography angiography, or OCTA, works quickly and does not require dye or invasive procedures. This method uses blood flow signals to take clear images of retinal and choroidal blood vessels layer by layer. It also avoids the problem of blurred vessel details caused by dye leakage (13, 14). Swept-source OCTA (SS-OCTA) uses a longer wavelength and faster scan speed than spectral-domain OCTA (SD-OCTA) used by Kaplan et al. (10). These improvements provide better tissue penetration and clearer images. SS-OCTA can show deep choroidal structures more distinctly. It also penetrates the retinal pigment epithelium more effectively and reduces motion artifacts. In this way, it solves many problems of SD-OCTA (13). The spider-like vascular configuration in our case was clearly visualized on en-face SS-OCTA with a level of detail not achievable with conventional ICGA. In terms of hemodynamic features, OCTA offered a clear definition of the vascular structure, with lower flow signals detected compared with the adjacent choroidal stroma. This finding corresponds to the slow-flow nature of venous channels and supports earlier reports of relatively stagnant flow in posterior venous drainage routes (4). In the present case, SS-OCTA enabled detailed visualization of the macular vortex vein’s configuration and drainage pattern, proved to be a useful imaging modality for recognizing this vascular variant.

In clinical practice, clinicians should correctly identify macular vortex veins as benign findings. This can help avoid misdiagnoses that may lead to unnecessary treatment. Gündüz et al. reported one case. A patient’s enlarged vortex vein ampulla was wrongly diagnosed as choroidal melanoma. The patient then had an enucleation that could have been avoided. This case highlights the importance of correctly recognizing these benign vascular structures (15). The primary differential diagnoses of macular vortex veins consist of choroidal neovascularization and choroidal macrovessel. The absence of leakage on ICGA and the characteristic vascular morphology with flow void on OCTA help distinguish PVVs from active neovascular disorders.

Consistent with previous reports, our patient remained asymptomatic with stable visual acuity. Ohno-Matsui et al. (16) reported an 11-year follow-up of a 10-year-old highly myopic child with a macular vortex vein, which gradually became more dilated and tortuous over time but with persistently stable vision. Cases reported by Kaplan et al. (10) were also asymptomatic. Nevertheless, PVVs are not always benign. Schouten et al. described a case of pathologic myopia with macular vortex vein complicated by choroidal neovascularization and Fuchs’ spot, suggesting that posterior venous drainage pathways may contribute to the development of neovascularization by affecting choroidal circulation (17). Therefore, long-term follow-up of such patients remains necessary.

Limitations of this report include the inherent drawbacks of a single-case, cross-sectional study. We cannot comment on the long-term evolution of this PVV or definitively determine whether it represents a congenital variant or acquired remodeling due to myopic progression. A longitudinal study by Lu et al. showed that 33.1% of PVVs exhibited branch attenuation, altered drainage routes, or even complete disappearance of the main trunk during a mean follow-up of 7.8 years, highlighting the importance of long-term follow-up in understanding their dynamic changes (18).

## Patient perspective

The patient reported that the floaters in her right eye were bothersome but did not affect her daily activities. She was initially concerned about the incidental finding in her left eye but was relieved to learn that it was a benign vascular variant that did not require treatment. She expressed appreciation for the thorough explanation of the imaging findings and understood the importance of annual follow-up.

## Data Availability

The raw data supporting the conclusions of this article will be made available by the authors, without undue reservation.
